# Rapid, efficient, and economical synthesis of PET tracers in a droplet microreactor: application to O-(2-[^18^F]fluoroethyl)-L-tyrosine ([^18^F]FET)

**DOI:** 10.1186/s41181-019-0082-3

**Published:** 2019-12-31

**Authors:** Ksenia Lisova, Bao Ying Chen, Jia Wang, Kelly Mun-Ming Fong, Peter M. Clark, R. Michael van Dam

**Affiliations:** 1 Physics in Biology and Medicine Interdepartmental Graduate Program, University of California, Los Angeles, Los Angeles, CA USA; 2 Crump Institute for Molecular Imaging, University of California, Los Angeles, Los Angeles, CA USA; 3 Department of Molecular & Medical Pharmacology, David Geffen School of Medicine, University of California, Los Angeles, Los Angeles, CA USA; 4 Department of Bioengineering, University of California, Los Angeles, Los Angeles, CA USA

**Keywords:** Radiochemistry, Microfluidics, FET, Amino acid imaging, Droplet synthesis, Molar activity, Pre-clinical imaging, Automation, Droplet microreactor

## Abstract

**Background:**

Conventional scale production of small batches of PET tracers (e.g. for preclinical imaging) is an inefficient use of resources. Using O-(2-[^18^F]fluoroethyl)-L-tyrosine ([^18^F]FET), we demonstrate that simple microvolume radiosynthesis techniques can improve the efficiency of production by consuming tiny amounts of precursor, and maintaining high molar activity of the tracers even with low starting activity.

**Procedures:**

The synthesis was carried out in microvolume droplets manipulated on a disposable patterned silicon “chip” affixed to a heater. A droplet of [^18^F]fluoride containing TBAHCO_3_ was first deposited onto a chip and dried at 100 °C. Subsequently, a droplet containing 60 nmol of precursor was added to the chip and the fluorination reaction was performed at 90 °C for 5 min. Removal of protecting groups was accomplished with a droplet of HCl heated at 90 °C for 3 min. Finally, the crude product was collected in a methanol-water mixture, purified via analytical-scale radio-HPLC and formulated in saline. As a demonstration, using [^18^F]FET produced on the chip, we prepared aliquots with different molar activities to explore the impact on preclinical PET imaging of tumor-bearing mice.

**Results:**

The microdroplet synthesis exhibited an overall decay-corrected radiochemical yield of 55 ± 7% (*n* = 4) after purification and formulation. When automated, the synthesis could be completed in 35 min. Starting with < 370 MBq of activity, ~ 150 MBq of [^18^F]FET could be produced, sufficient for multiple in vivo experiments, with high molar activities (48–119 GBq/μmol). The demonstration imaging study revealed the uptake of [^18^F]FET in subcutaneous tumors, but no significant differences in tumor uptake as a result of molar activity differences (ranging 0.37–48 GBq/μmol) were observed.

**Conclusions:**

A microdroplet synthesis of [^18^F]FET was developed demonstrating low reagent consumption, high yield, and high molar activity. The approach can be expanded to tracers other than [^18^F]FET, and adapted to produce higher quantities of the tracer sufficient for clinical PET imaging.

## Background

Positron emission tomography (PET) is a non-invasive molecular imaging tool based on the use of positron-emitting isotopes to track the position and dynamics of biologically relevant molecules in the body. PET provides high-sensitivity quantitative visualization of physiological parameters in vivo, such as metabolic rate, receptor density, gene expression, or blood flow, which makes it a versatile and potent tool for clinical diagnosis, treatment planning, treatment monitoring, as well as research (Aboagye et al. 2001; Ambrosini et al. 2009; Ciernik et al. 2003; Kitson et al. 2009; Phelps 2000).

Safe preparation of various target-specific PET tracers is a complex and expensive process, requiring skilled personnel operating expensive automated radiosynthesis equipment within radiation-shielded “hot cells”. With conventional apparatus, in which the chemistry is carried out in mL volume scales, relatively high reagent amounts (1 s to 10s of mg) are needed to achieve a sufficient concentration for good reaction yield in a short time, and for [^18^F]fluoride chemistry high amounts of radioactivity (10s of GBq) are needed to achieve high molar activity (Sergeev et al. 2018a). These factors contribute to inefficient use of resources in the preparation of small batches of tracers, such as needed for preclinical imaging, or for a single clinical PET scan, where much of the prepared batch would be wasted.

On the other hand, emerging microfluidic radiosynthesis methods require much lower amounts of reagents and radionuclide, and through substantially reduced instrument size and cost, have the potential to significantly reduce costs and resources needed for radiopharmaceutical production. Microscale reactions also tend to be faster and, due to the low precursor mass used, the crude products can be purified with simpler methods (e.g. analytical-scale high-performance liquid chromatography (HPLC) or cartridge instead of semi-preparative HPLC). These advantages are especially relevant for smaller batch production of PET tracers, but can also benefit the production of larger batches (Chao et al. 2018b).

Of the several different microfluidic approaches reported in the last decade (Elizarov 2009; Keng and van Dam 2015; Miller et al. 2010; Pascali and Matesic 2016; Rensch et al. 2013), microvolume reaction approaches offer the greatest potential for reagent and instrument reductions (Dooraghi et al. 2013; Elizarov et al. 2010; Iwata et al. 2018; Keng et al. 2016; Lebedev et al. 2012; Wang et al. 2017). A particular configuration we are exploring is performing reactions in microliter-sized droplets on simple Teflon-coated silicon microfluidic chips, which has advantages of simple operation, low-cost disposable chips, and a compact system size, which reduces the necessary shielding. Previous work has shown application of this method for the rapid and efficient synthesis of [^18^F]FDG and [^18^F]Fallypride (Wang et al. 2017). In this paper, we demonstrate further versatility of this approach by adapting the macroscale synthesis of O-(2-[^18^F]fluoroethyl)-L-tyrosine ([^18^F]FET) to this platform, and then use the produced [^18^F]FET for pre-clinical imaging.

[^18^F]FET is an amino acid PET probe (Wester et al. 1999), finding use in glioma imaging (Langen et al. 2017) as well as providing a route for protein labeling with fluorine-18 (Yanai et al. 2019). The radiosynthesis of [^18^F]FET from the commercially available precursor (2S)-O-(2′-tosyloxyethyl)-N-trityl-tyrosine-tert-butyl ester (TET) consists of a radiofluorination step followed by a hydrolysis step. The conventional synthesis typically results in good radiochemical yields (RCYs), ranging from 19 to 64% (Bourdier et al. 2011; Bouvet et al. 2012; Hamacher and Coenen 2002; Iwata et al. 2018; Lakshminarayanan et al. 2016). Some efforts have been made to carry out the synthesis in microfluidic format. Bouvet et al. performed the reaction in a commercial flow radiochemistry system using either microwave or heat activation of the reaction. An RCY of 50% was obtained with only 59 nmol of precursor in a 30 μL reaction in < 45 min (Bouvet et al. 2012), but to scale to larger production amounts (e.g., > 200 MBq) would require longer synthesis times and higher precursor amounts. Iwata et al. performed batch synthesis in 10–20 μL volumes (180–350 nmol of precursor) within a small glass vial by first loading a larger volume of methanolic solution containing [^18^F]fluoride and phase transfer catalyst, evaporating the solvent, then adding the small volume of precursor solution and performing the reaction (Iwata et al. 2018). Yields of up to 64 ± 11% (*n* = 3 ~ 6) were reported at scales of < 400 MBq. An automated procedure for this method was not described and may be challenging in practice due to the difficulty of manipulating small volumes in what is essentially a conventional apparatus.

We report a simple and rapid method for [^18^F]FET synthesis based on microvolume droplet approach. The probe production with this method results in high RCY and high molar activity using a very small amount of precursor and low starting activity. The low precursor amount enables purification via analytical, rather than semi-preparative, scale HPLC. This low-cost approach allowed us to carry out a large dynamic imaging study of up to 8 mice within a single day, thus demonstrating that the method will be a favorable option for pre-clinical studies of [^18^F]FET.

## Materials and methods

### Materials

#### Reagents and supplies

For the radiochemistry portion of this work, no-carrier-added [^18^F]fluoride was produced by the ^18^O(p, n)^18^F reaction from [^18^O]H_2_O (84% isotopic purity, Zevacor Pharma, Noblesville, IN, USA) in an RDS-112 cyclotron (Siemens; Knoxville, TN, USA) at 11 MeV using a 1 mL tantalum target with havar foil. Acetonitrile (MeCN; anhydrous, 99.8%), methanol (MeOH; anhydrous, 99.8%), ethanol (EtOH; 200 proof, > 99.5%), hydrochloric acid (HCl; 1 M), thexyl alcohol (2,3-dimethyl-2-butanol, 98%), trifluoroacetic acid (TFA, 99%), deionized (DI) water, phosphate buffered saline (PBS; pH 7.4) were purchased from Millipore Sigma (St. Louis, MO, USA). Saline (0.9% sodium chloride injection, USP) was obtained from Hospira Inc. (Lake Forest, IL, USA). All reagents were used as received without further purification. 18 MΩ water was obtained from a purification system (RODI-C-12BL, Aqua solutions, Inc., Georgia, USA). Tetrabutylammonium bicarbonate 0.075 M (TBAHCO_3_, > 99%), (2S)-O-(2′-tosyloxyethyl)-N-trityl-tyrosine-tert-butyl ester (TET, > 95%) precursor, O-2-fluoroethyl-L-tyrosine standard (FET-HCl, > 95%) were purchased from ABX GmbH (Radeberg, Germany).

To perform uptake assays, GS025 and GBM39 cells were kindly provided by Dr. David Nathanson (UCLA), the ParcB3 cells were provided by Dr. Peter Clark (UCLA), and the HCT-15 and HCC827 cells were purchased from ATCC (Manassas, VA, USA). Poly-L-lysine, protease inhibitor (cOmplete™), Hank’s balanced salt solution (HBSS; 10×), and fetal bovine serum (FBS),were purchased from Millipore Sigma (St. Louis, MO, USA). 96 well plates, 96 well filter plates, 0.25% trypsin, 100× penicillin-streptomycin (10,000 U/mL, Gibco™), RPMI-1640 medium (1×, Gibco™), GlutaMAX™ – I (100×, Life Technologies), Dulbecco’s Modified Eagle Medium (DMEM/F12), (100×), epidermal growth factor recombinant human protein (EGF), fibroblast growth factor recombinant human protein (FGF-Basic), heparin, and B27 supplement (50×) were purchased from Thermo Fisher (Waltham, MA, USA).

#### Analytical methods

A calibrated ion chamber (CRC 25-PET, Capintec, Florham Park, NJ, USA) was used to perform radioactivity measurements. For radio-thin-layer chromatography (TLC) analysis, TLC plates (Baker-flex silica gel IB-F sheets 2.5 × 7.5 cm, J.T. Baker, Phillipsburg, NJ) were spotted with 1 μL samples of the crude intermediate, crude final product, or purified final product, and were developed in 80% v/v MeCN in H_2_O, and then scanned with a radio-TLC scanner (miniGita star, Raytest, Inc., Wilmington, NC, USA), or with a Cerenkov luminescence imaging system (Dooraghi et al. 2013). Retention factors of the observed radioactive species were: 0.0 ([^18^F]fluoride), 0.3 ([^18^F]FET), and 0.8 (fluorinated intermediate).

Radio-HPLC analysis and purification was performed on an analytical-scale Smartline HPLC system (Knauer, Berlin, Germany) with 200 μL injection loop, a pump (Model 1000), degasser (Model 5050), a UV detector (Model 2500) and a radiometric detector (Bioscan B-FC-4000, Bioscan Inc., Washington DC, USA). Samples were separated using a C18 column (Luna, 4.6 × 250 mm, 5 μm, Phenomenex, Torrance, CA, USA) with guard column (SecurityGuard C18, Phenomenex) at a flow rate of 1 mL/min. UV absorbance was measured at 269 nm. Using 10% v/v EtOH in 18 MΩ H_2_O mobile phase, the expected retention time of [^18^F]fluoride was between 2 and 3 min, and around 5 min for [^18^F]FET. The fluorinated intermediate was eluted off the column using 100% MeCN.

#### Microfluidic systems

Radiochemistry was performed in droplet format on the surface of microfluidic chips comprising a silicon wafer with a patterned Teflon AF coating. The detailed fabrication was previously reported (Wang et al. 2017). A combination of hydrophobic (Teflon) and hydrophilic (exposed silicon wafer) regions allows liquid droplets to be manipulated or maintained in a desired location to perform reactions.

**Fig. 1:**
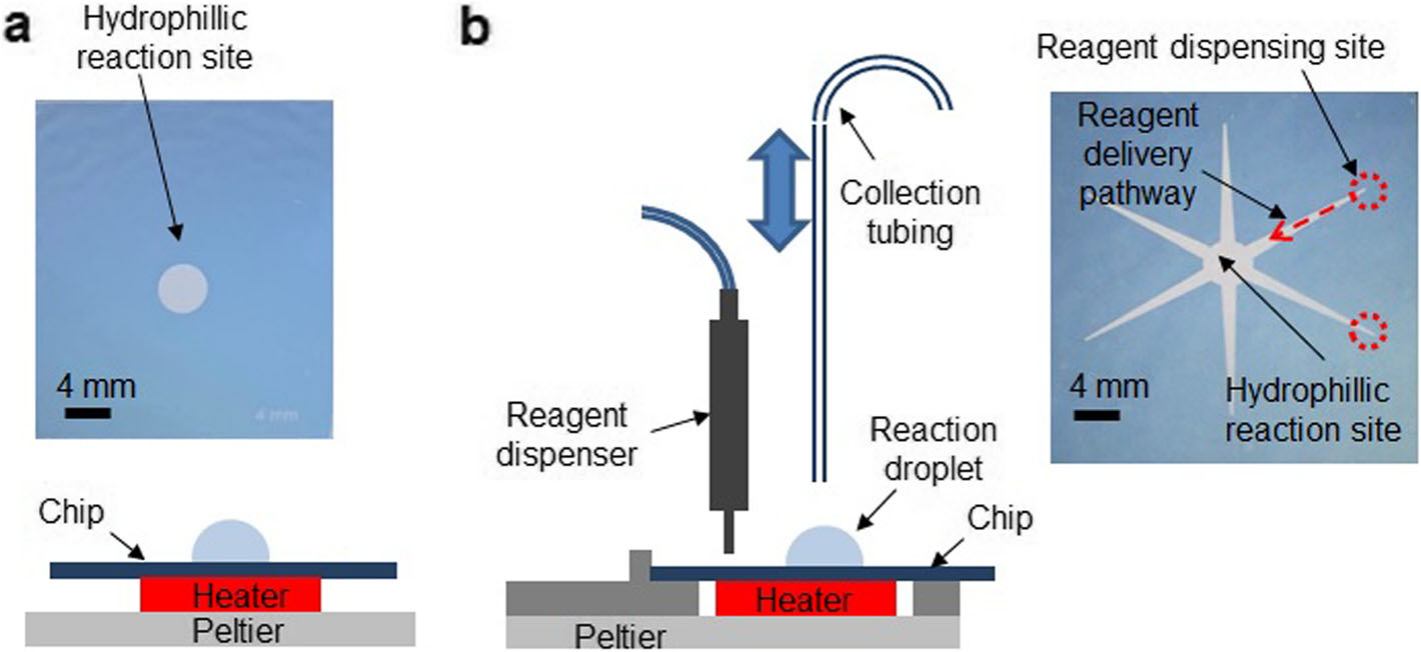
Side view schematic of manual **a** and automated **b** microvolume synthesis platform, and top view photographs of corresponding chips used

One type of chip, used for the synthesis optimization studies, had a 4 mm circular hydrophilic region (i.e. Teflon coating etched away) serving as a reaction site (Fig. 1a). During use, the chips were mounted to a temperature control platform comprising a ceramic heater affixed to a Peltier device, which was in turn mounted on a heat sink with a fan. A thin layer of thermal paste was present between all device components to ensure good thermal contact. Reagents were loaded and product was collected manually via pipette with 10 or 200 μL tips.

Another type of chip, used for automated synthesis, had six radial tapered hydrophilic pathways leading toward a central hydrophilic reaction site (Fig. 1b). The chip was similarly mounted to a temperature-control platform, but reagents were added via electronically controlled piezoelectric actuators around the periphery of the chip and crude product was collected by a retractable needle. The tapered pathways spontaneously transport reagent droplets from the periphery to the center of the chip. Complete details of this setup were reported previously (Wang et al. 2017).

### Methods

#### Microscale radiosynthesis and purification of [^18^F]FET

**Fig. 2:**
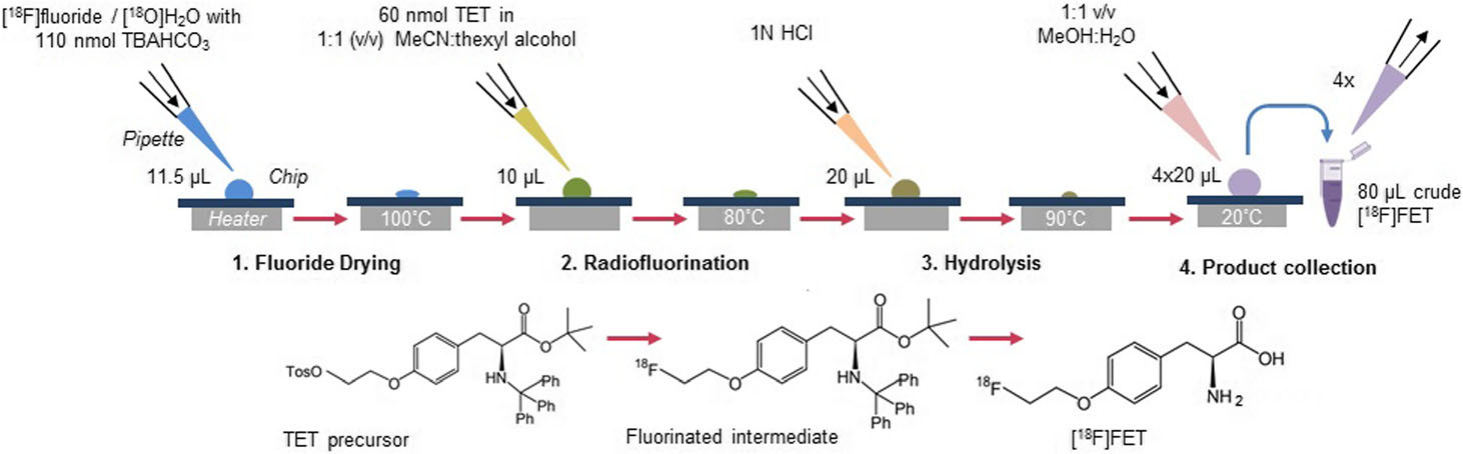
Synthesis scheme for microvolume production of [^18^F]FET using manual synthesis platform

The microscale synthesis was adapted from previously described macroscale protocols (Bourdier et al. 2011; Hamacher and Coenen 2002) by scaling down reagent volumes (Fig. 2). Cyclotron produced [^18^F]fluoride (37–740 MBq in ~ 10–20 μL) was mixed with 110 nmol of TBAHCO_3_ (i.e., 1.5 μL of 75 mM solution), deposited on the chip and then evaporated to dryness at 100 °C. After cooling to 30 °C, 10 μL of 6 mM TET (60 nmol) in 1:1 v/v MeCN:thexyl alcohol was added to the chip. The reaction mixture was heated at 90 °C for 5 min, and then cooled to 30 °C. Next, 20 μL of 1 M HCl was added and the deprotection reaction was performed by heating to 90 °C for 3 min. The crude product was recovered by adding 20 μL of 1:1 v/v MeOH:H_2_O and collecting from the chip. The collection process was repeated a total of 4 times to ensure high recovery of the crude product. After synthesis, the product was diluted to 150–175 μL using HPLC mobile phase (10% v/v EtOH in 18 MΩ water) and purified via analytical-scale radio-HPLC. The product peak was collected (typically 1.0–1.5 min duration) into a sterile glass vial. Solvent was evaporated by heating the vial to 120 °C with an oil bath and applying a nitrogen stream above the surface of the solvent. When dry (typically after 10–15 min), the [^18^F]FET was then resuspended either in sterile saline for in vivo imaging, or pH 7.4 phosphate-buffered saline (PBS) for cell uptake experiments. Numerous intermediate measurements were taken during synthesis to carefully analyze its performance (see details in Electronic supplementary material (ESM), Additional file 1: Sects. 1–2).

#### In vitro probe uptake

In vitro uptake of [^18^F]FET was compared across two glioblastoma cell lines (GS025, GBM39), a prostate cancer cell line (ParcB3), a lung cancer cell line (HCC827), and a colon cancer cell line (HCT-15). The GS025, GBM39, and ParcB3 suspension cells were grown in stem cell media, and the HCC827 and HCT-15 adherent cells were grown in supplemented RPMI. The suspension cells were plated into 96-well plates and adherent cells into 96-well filter plates at 150, 000 cells/mL concentration in 1x HBSS. [^18^F]FET was diluted to a concentration of 370 Bq/μL with either PBS (for uptake experiments) or PBS containing 5 mM FET (for blocking experiments). Cell uptake experiments were performed by adding 100 μL [^18^F]FET (37 kBq) to each of a set of cell wells (*n* = 4), and blocking experiments (to confirm specificity) were performed by adding 100 μL [^18^F]FET (37 kBq) with FET (500 nmol) to each of a set of cell wells (*n* = 4). The cells were incubated at 37 °C for 10 min, then transferred into individual gamma counter tubes and sample radioactivity was measured on a gamma counter (WIZARD 3″ 1480, Perkin Elmer, Waltham, MA, USA). The uptake values were normalized to total protein amounts for each sample (complete details of the procedure are provided in the ESM, Additional file 1: Sect. 3). The statistical significance of the values was validated by a two-tailed T test (*p* < 0.05).

#### In vivo preclinical imaging

Male NOD *scid* gamma (NSG) mice ~ 7 week-old were obtained from the UCLA Department of Radiation Oncology. These mice (*n* = 10) were engrafted with 0.5 × 10^6^ HCC827 cells suspended in a 1:1 (v/v) mixture of supplemented RPMI media and Corning® Matrigel® Basement Membrane Matrix (Corning Life Sciences) in the left and right shoulders.

To perform dynamic PET imaging, mice were kept under 2% isoflurane anesthesia during the tracer uptake for 60 min. Mice were injected with 1.5–3.1 MBq of the tracer, and were scanned 4 at a time using the recently developed HiPET scanner (Gu et al. 2017). The first study was performed with 4 mice injected with probe of different molar activities in a range of 1.5–36 GBq/μmol (*n* = 1 each). The second study with 8 mice covered molar activities ranging from 0.4–48 GBq/μmol (*n* = 2 each) (see Additional file 1: ESM, Sect. 4 for details). The concentration of FET in blood was estimated to range between 0.02 and 3.5 *μ*M assuming 2 mL average mouse blood volume. All mice received 10 min CT scans (CrumpCAT (Taschereau et al. 2014)) following the PET imaging experiment. After PET/CT image registration, regions of interest (ROI) were drawn with AMIDE version 1.0.5 software, and the results were analyzed by comparing mean intensity values of the tumors and other regions across different time points (12 frames of 5 min each) (details on image analysis are described in ESM, Additional file 1: Sect. 5**)**.

## Results

### Microscale [^18^F]FET synthesis optimization and automation

To adapt the 2-step synthesis of [^18^F]FET from the macroscale to the microscale, the precursor and base quantities were initially scaled down nearly 300–490-fold from values reported in conventional synthesis (Bourdier et al. 2011; Hamacher and Coenen 2002), i.e. to 75 nmol of TBAHCO_3_ (1 μL, 0.075 M) and 30 nmol of the TET precursor in 20 μL. We used TBAHCO_3_ rather than K_222_/K_2_CO_3_ (Bourdier et al. 2011; Iwata et al. 2018) based on the suggestion by Hamacher and Coenen (2002), who observed higher yields due to the lower basicity and reduced competing elimination reaction. One significant change we made was altering the fluorination reaction solvent. The syntheses reported by Hamacher and Coenen (2002), Bourdier et al. (2011) and Lakshminarayanan et al. (2016) all used MeCN as the fluorination solvent, but in the droplet format, we found that the MeCN evaporated very quickly, resulting in poor yields. After exploring several solvent combinations that have been previously reported in droplet reactions (Keng and van Dam 2015), a 1:1 v/v mixture of MeCN and thexyl alcohol was selected. During early syntheses the fluorination temperature was set at 80 °C, slightly lower than what has been reported in conventional syntheses (i.e., 85 °C (Hamacher and Coenen 2002) or 100 °C (Bourdier et al. 2011)) to further mitigate evaporation, and the reaction time was set for 5 min. Under these conditions, fluorination yield was 36 ± 7% (*n* = 4).

**Fig. 3:**
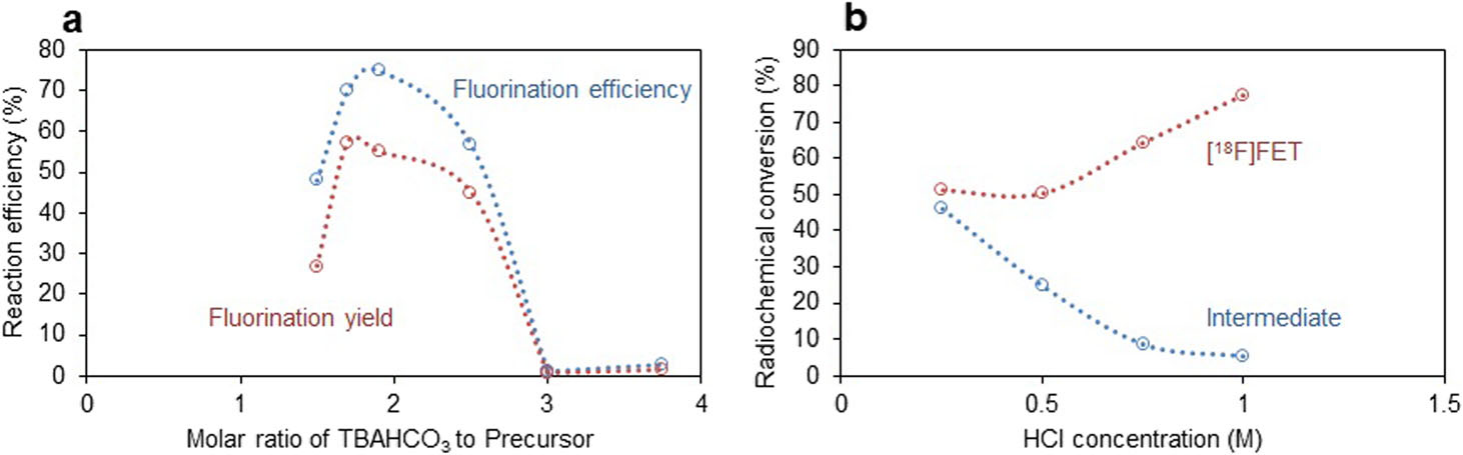
**a** Effect of base to precursor ratio on fluorination efficiency and fluorination yield (*n* = 1 for each data point). Syntheses carried out at 80 °C for 5 min with 30 nmol or 60 nmol of precursor. **b** Effect of deprotectant (10 μL HCl) concentration on deprotection reaction at 90 °C for 3 min (*n* = 1 for each condition). Synthesis performed with 60 nmol precursor and 110 nmol TBAHCO_3_ at 90 °C for 5 min

**Fig. 4:**
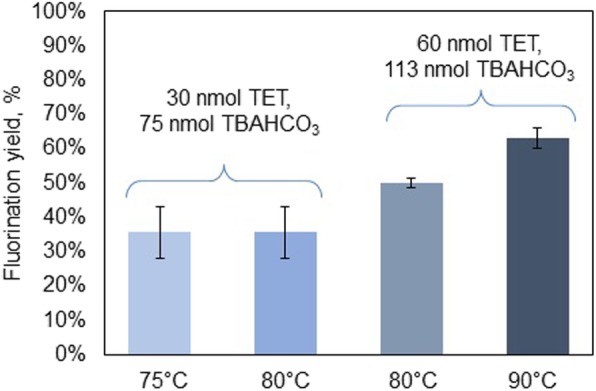
Results of initial optimization of fluorination conditions. Error bars represent standard deviations (*n* = 4)

Investigation of the ratio of base to precursor (Fig. 3a) indicated that the originally chosen ratio (~ 2.5) was close to optimal: a steep drop in fluorination efficiency was observed for base to precursor ratios below 1.7 and higher than 2.5. When fluorinating with 110 nmol of TBAHCO_3_ per 60 nmol of precursor (1.9 ratio) at 80 °C the fluorination yield reached 50 ± 1% (*n* = 4). Increasing the temperature to 90 °C further improved the fluorination yield to 63 ± 3% (*n* = 4) (Fig. 4). Lower reaction temperature (75 °C) resulted in similar yield as the 80 °C reaction, though solvent evaporation was slightly reduced (Fig. 4). Later, the reaction volume was reduced to 10 μL keeping the same amount (60 nmol) of precursor per reaction to make it more compatible with chip chemistry, however no significant change in reaction yield was observed.

For the deprotection step, we initially attempted to use TFA as reported by Hamacher and Coenen (2002) and Bouvet et al. (2012); however, we observed rapid evaporation of TFA and low deprotection efficiency. We then explored the use of HCl, as reported by Bourdier et al. (2011) and Lakshminarayanan et al. (2016). Using a deprotection reagent volume of 10 μL heated for 3 min at 90 °C, we explored the effect of different HCl concentrations (Fig. 3b). Higher concentrations resulted in more complete deprotection of the intermediate. The use of 10 μL of 1.0 M HCl was sufficient to deprotect most of the intermediate (~ 94%). Increasing the volume from 10 to 20 μL led to improved hydrolysis and was used in all subsequent experiments. Conveniently, the acid nearly fully evaporates during the hydrolysis step leaving only trace amounts of liquid, obviating the need for neutralization.

The manual synthesis of the crude product, under optimized conditions, required 24 ± 2 min (*n* = 4). The collection efficiency was 64 ± 5% (*n* = 4) and radiochemical conversion of [^18^F]FET was 92 ± 4% (*n* = 4) resulting in crude RCY of 59 ± 7% (*n* = 4). Fluorination yield was estimated to be 62 ± 8% (*n* = 4), and hydrolysis efficiency was 96 ± 2% (*n* = 4). Only 1.3 ± 0.5% (*n* = 4) of the starting activity was attributed to residual activity on the chip after collection of the crude product, though an additional loss of 35 ± 6% (*n* = 4) was observed, potentially corresponding to loss of unreacted [^18^F]fluoride in the form of [^18^F]HF during the acidic deprotection step.

Production of [^18^F]FET for imaging was performed using this manual protocol, followed by purification by analytical HPLC (~ 5 min) and formulation (10–15 min), resulting in an overall synthesis time of 40 min. The loss during purification and formulation was 7 ± 3% (*n* = 4) and overall decay corrected RCY was 55 ± 7% (*n* = 4). The identity of the purified product was confirmed via analytical radio-HPLC by co-injection with the reference standard. Radiochemical purity of the final product as determined via radio-HPLC was > 98%. Molar activity was 48–119 GBq/μmol at the end of synthesis.

We also performed the synthesis using the automated droplet radiosynthesizer (i.e. with the passive transport microfluidic chips) and observed a crude decay-corrected RCY of 54 ± 6% (*n* = 5) (a detailed comparison of the performance of the manual and automated droplet synthesis processes is summarized in Table 1). In general, the performance was very similar, the main difference being slightly lower collection efficiency with the automated procedure. An advantage of the automated synthesis is that the synthesis of the crude product was completed in a shorter time (5 min less).

**Table 1:** Summary of performance of microdroplet synthesis of [^18^F]FET with optimized manual operation or automated operation. All values are decay-corrected unless otherwise specified

	Manual (*n* = 4)	Automated (*n* = 5)
Collection efficiency (%)	64 ± 5	59 ± 10
Residual chip activity (%)	1.3 ± 0.5	3 ± 1
Volatile activity loss (%)	35 ± 6	38 ± 11
Fluorination yield (%)	62 ± 8	59 ± 10
Radiochemical conversion to FET (%)	92 ± 4	93 ± 6
Deprotection efficiency (%)	96 ± 2	93 ± 6
Crude RCY (%)	59 ± 7	54 ± 6
Crude synthesis time (min)	24 ± 2	19 ± 2
Crude RCY, non-decay-corrected (%)	51 ± 6	48 ± 5

### In vivo imaging at varying molar activities of [^18^F]FET

As a demonstration of the ability to perform a preclinical imaging study with [^18^F]FET produced using the microscale method, we prepared [^18^F]FET of different molar activities to investigate the impact on in vivo imaging. It has been previously seen with imaging of [^18^F]Fallypride that molar activity can significantly affect the PET imaging contrast in the striata of the brain (Sergeev et al. 2018b), whereas variations in molar activity of [^18^F]FDOPA were reported not to impact the imaging of neuroendocrine tumors (Kuik et al. 2015). [^18^F]FET is one of the major fluorine-18 labeled amino acids used in glioma imaging, grading and therapy planning. [^18^F]FET is an L-tyrosine analogue, and it helps to visualize amino acid transport activity that is upregulated in many growing tumors (Langen et al. 2006, 2017).

To perform experiments, samples with different molar activities were prepared from a single batch of [^18^F]FET. The batch was divided into four aliquots, then each aliquot was spiked with different amounts of the reference standard and saline to achieve different molar activity values with the same radioactivity concentration (details of the preparation are included in the ESM, Additional file 1: Sect. 4).

**Fig. 5:**
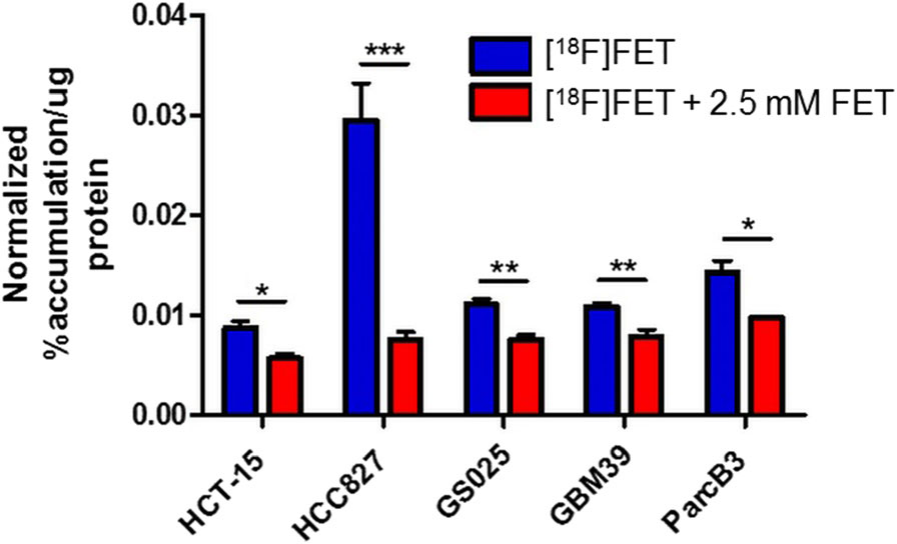
Accumulation of [^18^F]FET in different cell lines. Error bars represent standard deviation (*n* = 4). (*) p < 0.05, (**) *p* < 0.01, (***) *p* < 0.001. The red bars indicate incubation with both [^18^F]FET and 2.5 mM FET reference standard to establish specificity

The cell uptake comparison among few different cell lines had shown that the lung cancer cell line HCC827 had a significantly higher probe uptake than any of the other cell lines tested (GS025, GBM39, ParcB3, HCT-15) (Fig. 5) and was used for in vivo study. Subcutaneous tumor HCC827 xenograft models had reached sufficient tumor size for imaging (~ 4 mm diameter) after 36 days when an initial imaging experiment was performed (ESM, Additional file 1: Fig. S3), followed by another study at 50 days post-implantation (ESM, Additional file 1: Fig. S4). Dynamic PET/CT scans were performed with injections of different molar activities. In all cases, the signal in the blood was high after injection and decreased over time. Muscle and tumor uptake rose gradually and plateaued at ~ 30 min, remaining nearly constant until the end of the scan. No bone uptake was observed in scans, confirming the lack of in vivo defluorination. Combined dynamic imaging data is summarized in ESM, Additional file 1: Fig. S5–7.

**Fig. 6:**
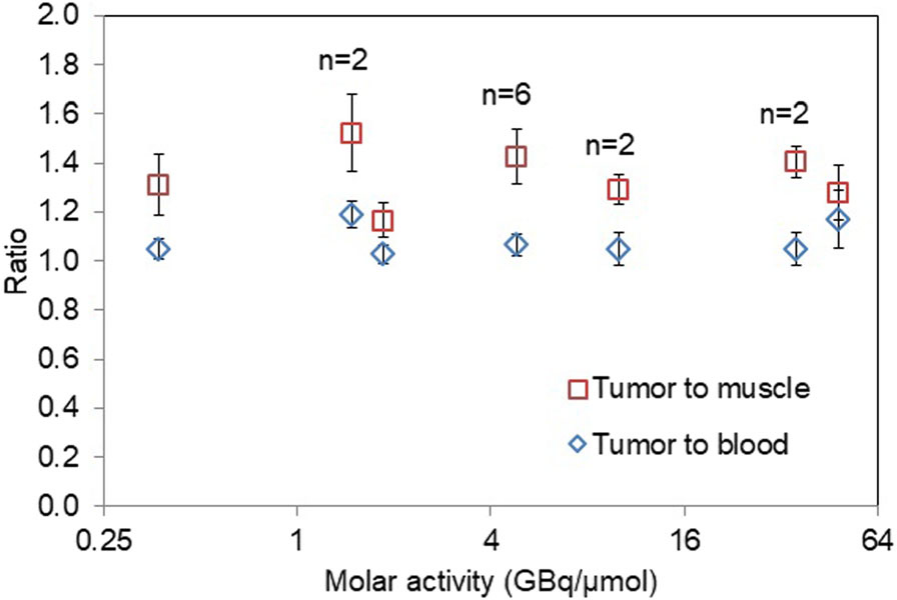
Tumor to muscle and tumor to blood ratios averaged for all tumors within the same molar activity value group (*n* = 4 except as otherwise indicated) and averaged over the dynamic imaging data from 30 to 60 min. Error bars represent standard deviation

The tumor to blood ratio increased during the first 15–20 min and then remained nearly constant for the rest of the scan, while the tumor to muscle ratio remained nearly constant throughout the scan (Additional file 1: Fig. S7). Qualitatively, it is apparent there is no strong correlation between the tumor uptake ratios and the molar activity values. Tumors imaged at low molar activity were as easily visible as tumors imaged at high molar activity of the injected probe. The uptake ratios averaged during the final 30–60 min of the scans summarized for different molar activity values did not exhibit any correlation either (Fig. 6). The statistically insignificant correlation between uptake ratios and molar activity was confirmed using a Spearman correlation test (r_s_ = − 0.3 for tumor to muscle ratio, r_s_ = 0.1 for tumor to blood ratio).

## Discussion

### Microscale synthesis

The microscale synthesis described here was performed quickly, reliably and in high yield, allowing production of the tracer for pre-clinical studies. A comparison of the performance of the microvolume synthesis compared to conventional synthesis is included in Table 2. The consumption of reagents was reduced drastically (> 150 × less precursor) compared to conventional methods, while still achieving comparable RCY. Though optimization runs (requiring numerous intermediate measurements), and batches for imaging (where molar activity adjustments were needed at the end) took longer to prepare, the fully-automated microvolume synthesis can be completed in 35 min (19 min synthesis + 6 min purification via analytical-HPLC + 10 min formulation). This is significantly faster than macroscale synthesis methods, and is a significant advantage when considering non-decay-corrected RCY. The short synthesis time originates from the smaller reaction volume, which enables faster temperature change and shorter solvent removal times, as well as from the low precursor mass, which enables the use of analytical scale HPLC purification rather than semi-preparative. Interestingly, the droplet method also resulted in shorter synthesis time and higher yield compared to recent reports of [^18^F]FET synthesis in smaller volumes (10s of microliters) using manual liquid manipulation or flow-through reactors (Table 2).

**Table 2:** Comparison of performance of the microvolume droplet synthesis of [^18^F]FET and published results using conventional methods

	This work	(Hamacher and Coenen 2002)	(Bourdier et al. 2011)	(Lakshminarayanan et al. 2016)	(Iwata et al. 2018)	(Yanai et al. 2019)	(Bouvet et al. 2012)
Reaction format	Droplet	Conventional	Conventional	Conventional	Small volume in vial	Small volume in vial	Flow-through / capillary
Reactor type	Droplet microreactor	Custom FDG module	TracerLab FX_FN_	Modified GE TracerLab FX-C	300μL Reacti-vial	300μL Reacti-vial	Advion NanoTek® capillary reactor
Precursor amount (nmol)	60	14800	9000	13280	180-350	350	59^d^
Starting activity (GBq)	0.4±0.1 (*n*=4)	N/R	18-41 (*n*=22)	N.R.	<0.4	0.95-2.6 (*n*=9)	0.005-0.2^d^ (*n*=?)
Reaction volume (μL)	10	500	2000	1000	10-20	20-30	20
Overall RCY (non decay-corrected, %)	55±7 (*n*=4)	33-36 (*n*=?)	35±5 (*n*=22)	19±1 (*n*=?)	N. R. ^c^ (*n*=3~6)	38±6 (*n*=9)	38 (*n*=?)
Synthesis time ^a^ (min)	40	80	63	N.R.	N.R.	60	<45
Molar activity (GBq/μmol)	56-140	>18	>90	N.R. b	N.R.	570±240 (*n*=9)	N.R.

*N.R*. not reported

^a^Synthesis time includes purification and formulation, except Bouvet et al. which does not include formulation

^b^The paper assumes the molar activity value of the tracer is the same as the [^18^F]fluoride in the irradiated target, which is not valid

^c^The decay-corrected RCY was reported as 34–64%, but no synthesis time was given, so an estimate of the non-decay corrected RCY could not be made.

^d^Unlike the other reaction formats, increasing the scale in a flow-through reactor requires increased reagent volumes and increased precursor consumption

Under optimized conditions, a batch of [^18^F]FET suitable for preclinical imaging throughout the day (e.g. 37–110 MBq; assuming 0.93–7.4 MBq per injection for 5–10 mice) could be produced on the microscale platform starting with only 110–330 MBq of [^18^F]fluoride. Limiting the activity to relatively low levels in this manner could have significant advantages for shielding the apparatus (i.e., thinner shielding would be adequate) and possibly operating the synthesis outside of a hot cell.

The droplet synthesis (even with starting activities lower than 0.74 GBq) resulted in high molar activities, comparable to the values achieved on macroscale synthesizers starting with > 30 GBq of fluoride-18. It should be appreciated that, when the starting activity is scaled down in macroscale radiosynthesizers, one observes a linear decrease in the resulting molar activity (Sergeev et al. 2018a). Thus, high amounts of starting activity must often be used in macroscale synthesizers, even if only a relatively small amount of the final tracer is needed. Compared to microscale synthesis, this can result in higher cost of the radioisotope, and the need for considerably more shielding to work with the higher activity levels.

Overall, the microvolume synthesis of radiopharmaceuticals has a number of advantages over conventional scale radiosynthesizers such as more compact apparatus, reduced shielding, rapid synthesis, high yield, and efficient use of radioisotope. These advantages have the potential to drive down the costs of materials and infrastructure, which can be a significant benefit for limited resource settings or preclinical tracer production. Another advantage – low precursor consumption – not only helps to simplify the purification step, but can also represent a significant cost reduction, especially for tracers with expensive precursors, or in situations where precursor is scarce, such as the development of novel tracers or optimization of synthesis protocols. While the strengths of this technology are in reducing costs of small batches of tracers, e.g. to support in vitro or preclinical studies, various microfluidic technologies are constantly improving and expanding their applications in the radiopharmacy field, and could also lead to improvements in the efficiency of clinical PET tracer production in the future (Pascali et al. 2013), such as enabling the production of additional tracers with minimal need for extra space or capital.

### [^18^F]FET imaging

Over the range tested (0.37–48 GBq/μmol), the molar activity had no statistically significant effect on imaging of subcutaneous HCC827 tumors. [^18^F]FET accumulates in cells following transport by Na^+^-dependent and -independent amino acid transporters and is not incorporated into proteins over the time course of the imaging experiments (Heiss et al. 1999; Langen et al. 2003). The results suggest that the [^18^F]FET transporters on the lung cancer cell line HCC827 do not become saturated within the range of molar activity values tested. Though the in vitro experiments suggest that the transporters can be “saturated” with sufficient concentration of FET in the media (i.e., 2.5 mM in the case “spiked” with FET; 0.15 μM in the non-spiked condition), the estimated concentration of FET in blood during the in vivo experiments was much lower (i.e. 3.5 μM for the lowest molar activity of 0.37 GBq/μmol, assuming 2 mL blood volume).

## Conclusion

In this work the synthesis of [^18^F]FET was adapted to an automated microdroplet synthesis platform (Wang et al. 2017). The product was obtained in high RCY of up to 55 ± 7% (*n* = 4, decay-corrected) after purification, in sufficient quantities to perform a demonstration of a multi-animal dynamic PET imaging study, and could readily be scaled to higher amounts using radionuclide concentration methods (Chao et al. 2018a). Synthesis time was shorter than conventional approaches, precursor consumption was reduced by two orders of magnitude, and the synthesis could be performed with a very small apparatus. The low precursor consumption enabled faster and simpler purification (i.e., analytical HPLC instead of semi-preparative HPLC), and, for tracers with expensive precursors, could help to reduce the synthesis cost. The molar activity was high (48–119 GBq/μmol at the end of synthesis), even when starting with activities as low as 0.3 GBq. Though low molar activity of [^18^F]FET, (down to 0.37 GBq/μmol) did not appear to adversely affect imaging of subcutaneous tumors in this study, the ability to produce small batches with high molar activity may be important in other applications of this or other tracers.

## Supplementary information

**Additional file 1.** Supplemental information.

## Data Availability

All data generated or analyzed during this study are included in this published article and its supplementary information files. Additional raw measurements are available from the corresponding author on reasonable request.

## Abbreviations

[^18^F]FET: O-(2-[^18^F]fluoroethyl)-L-tyrosine
CT: Computed tomography
ESM: Electronic supplementary material
HPLC: High-performance liquid chromatography
NSG: NOD *scid* gamma
PET: Positron emission tomography
RCY: Radiochemical yield
TLC: Thin-layer chromatography
