# Supplementary material for: Rapid, efficient, and economical synthesis of PET tracers in a droplet microreactor: application to O-(2-[18F]fluoroethyl)-L-tyrosine ([18F]FET)
Source: EJNMMI Radiopharm Chem. 2019 Dec 31;5:1. doi: 10.1186/s41181-019-0082-3 (PMC6938530; doi:10.1186/s41181-019-0082-3)
Supplement: Supplementary file 1 — Additional file 1. Supplemental information. [file 41181_2019_82_MOESM1_ESM.pdf]

Additional file 1: Supplemental information

Rapid, efficient, and economical synthesis of PET tracers in a droplet microreactor: application to O-(2-[^{18}F]fluoroethyl)-L-tyrosine ([^{18}F]FET)

Ksenia Lisova[1,2,3], Bao Ying Chen[1,2,3], Jia Wang [2,3,4], Kelly Mun-Ming Fong[2,3], Peter M. Clark[1,2,3], R. Michael van Dam[1,2,3,4*]

[1]Physics in Biology and Medicine Interdepartmental Graduate Program; [2]Crump Institute for Molecular Imaging; [3]Department of Molecular & Medical Pharmacology, [4] Department of Bioengineering, UCLA, Los Angeles, CA, USA

[*]Corresponding author:
R. Michael van Dam, Email: mvandam@mednet.ucla.edu
Address: 4323 CNSI, 570 Westwood Plaza, Los Angeles, CA 90095, USA; Phone: +1-310-206-6507

Contents

1 Analysis of microscale synthesis performance 1
1.1 Activity measurements 1
1.2 Reaction efficiency 2
1.3 Purification and formulation 2
1.4 Molar activity 2
2 Calibration curve for molar activity determination 3
3 Cell uptake measurements 4
4 Preparation of samples with different molar activities 5
5 *In vivo* imaging in HCC827 xenograft mice 6
6 References 10

1 Analysis of microscale synthesis performance

1.1 Activity measurements

During the development and optimization of the microscale synthesis, numerous intermediate measurements were taken to thoroughly analyze the performance. All radioactivity values were decay-corrected to a common reference time. The starting activity loaded on the chip was determined by calculating the difference in activity of a source vial and pipette tip before and after addition to the chip.

The activity of the crude reaction product recovered from the chip, and the residual activity on the chip after the collection step were measured as well (The residual activity on the pipette tip used for collection was found to be negligible). Collection efficiency was calculated as the activity of the recovered crude product divided by the starting activity. Unaccounted activity loss was defined by subtracting the collected activity and the residual activity on chip from the starting activity and expressing as a fraction of starting activity.

1.2 Reaction efficiency

To assess reaction efficiency, a small portion of the collected crude product was analyzed via radio-TLC or/and radio-HPLC.

During initial optimization, only the fluorination step was performed resulting in formation of the fluorinated intermediate (with intact protecting groups). After collection of the crude mixture from the chip, a small (1 μL) sample was analyzed via radio-TLC, which exhibited two peaks ([^{18}F]fluoride and the intermediate). Fluorination efficiency was found from radio-TLC chromatogram by dividing the peak area corresponding to the intermediate by the sum of areas of all peaks. Fluorination yield was computed by multiplying the fluorination efficiency by collection efficiency.

When analyzing samples after performing both fluorination and hydrolysis, a small (1 μL) sample of the crude reaction mixture was taken for TLC analysis, which exhibited three peaks ([^{18}F]fluoride, [^{18}F]FET, and the intermediate). The radiochemical conversions (RCCs) for the intermediate and [^{18}F]FET were computed as the area under the corresponding peak divided by the area under all peaks. The fluorination yield was computed as the sum of the intermediate and [^{18}F]FET RCCs, multiplied by the collection efficiency. The hydrolysis efficiency was determined by dividing the peak area corresponding to [^{18}F]FET by the sum of the intermediate and [^{18}F]FET peak areas. Finally, the crude RCY was calculated by multiplying the [^{18}F]FET RCC by the collection efficiency.

1.3 Purification and formulation

Purification efficiency was calculated as the activity of the collected product after HPLC purification divided by the expected amount of pure product (i.e., the total activity of the crude product multiplied by the radiochemical purity (RCP)). To determine RCP, the crude product was analyzed via both radio-TLC and radio-HPLC. The radio-TLC chromatogram was used to compute the fraction of [^{18}F]fluoride incorporated into other species, computed as the total area of all non-fluoride peaks divided by the total area of all peaks. The radio-HPLC chromatogram was used to determine the fraction of non-fluoride peaks attributable to [^{18}F]FET. This fraction was computed as the peak area of [^{18}F]FET divided by the total area of all non-fluoride peaks. The RCP was computed as the radio-TLC fraction multiplied by the radio-HPLC fraction. In general, the radio-HPLC chromatogram obtained during the purification process could be used for this analysis (unless peaks were saturated).

The formulation efficiency was calculated as a ratio of the activity of final formulated product to the initial activity of the purified [^{18}F]FET.

1.4 Molar activity

The molar activity was determined using analytical radio-HPLC directly from the purification chromatogram. It was calculated by dividing the activity of [^{18}F]FET injected into HPLC by molar quantity of FET injected. The molar quantity of FET was determined using the area under the FET peak in the UV chromatogram and a calibration curve (ESM, section 2).

2 Calibration curve for molar activity determination

Since several different wavelengths for UV detection of FET in HPLC have been reported in literature (i.e., 200 nm (Lakshminarayanan et al. 2016), 220 nm (Bourdier et al. 2011), 280 nm (Iwata et al. 2018), we first measured the FET absorbance spectrum to determine a suitable wavelength. The absorbance of FET reference standard (500 µM) was scanned across a range of wavelengths 200-350 nm using a microfluidic UV absorbance detection device (Ly et al. 2018) and the absorbance spectrum of the solvent was subtracted. The resulting spectrum is shown in **Figure S1**. We did not select the maximum at 222 nm due to the high noise level, but instead chose the maximum at 269 nm.

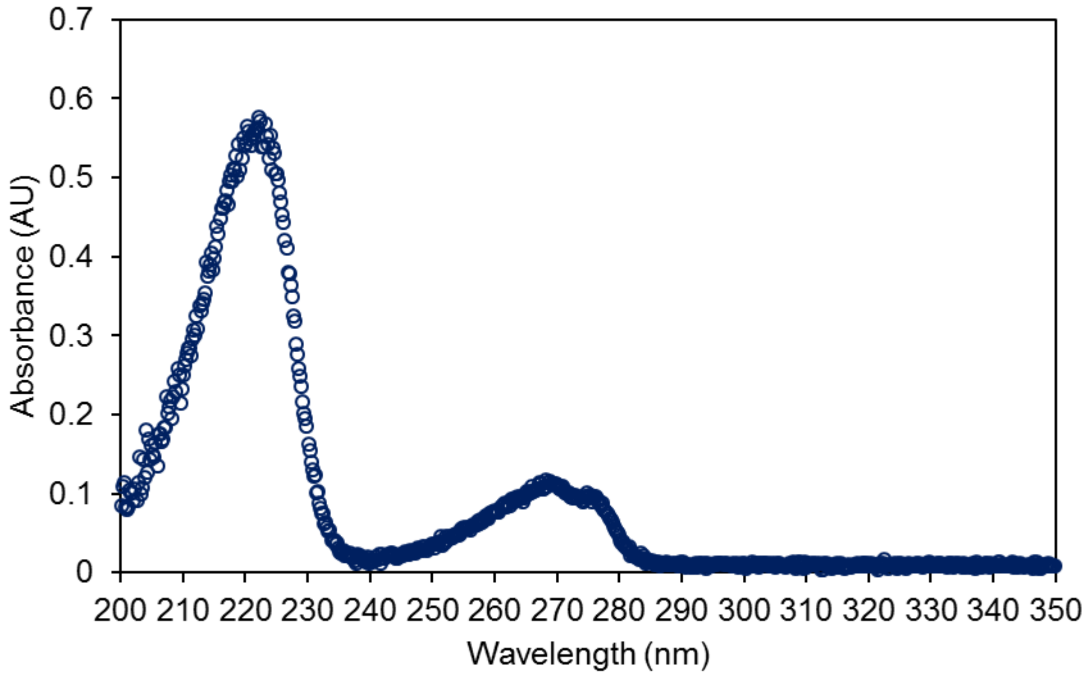

Fig. S1: UV absorbance spectrum of FET reference standard.

A calibration curve was then created for use in molar activity determinations. Different volumes of 50 µM FET reference standard (in HPLC mobile phase, i.e. 10% v/v EtOH:H_2O) were injected into the analytical HPLC, i.e. 10 µL (0.5 nmol), 20 µL (1 nmol), 30 µL (1.5 nmol), 40 µL (2 nmol), 50 µL (2.5 nmol) and 100 µL (5 nmol). The areas of the result peaks in the HPLC chromatogram were plotted as a function of molar amount of FET, and a linear least-squares fit was calculated **(**R^2 = 0.9997; **Figure S2**).

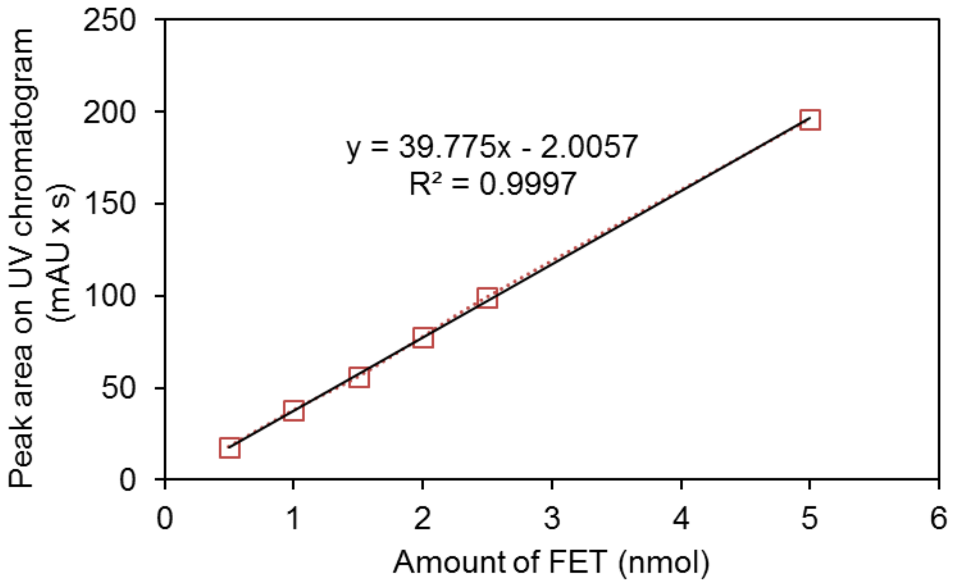

Fig. S2: Calibration curve for FET molar activity determination.

3 Cell uptake measurements

The fully-supplemented RPMI was prepared from 500 mL RPMI, 5 mL GlutaMAX, 50 mL FBS, 5 mL Penicillin-Streptomycin. Stem cell media was prepared in 500 mL of DMEM/F12 with 5.5 mL of GlutaMAX, 5.5 mL of Penicillin-Streptomycin and 10 mL of B27 supplement. 400× HEF mixture contained 2 mg/mL Heparin (0.22 µm filtered), 20 µg/mL EGF, 8 µg/mL FGF. 400× HEF mixture was diluted at 25 µL per 10 mL of culturing media, and was added to the cells every 5 days during culturing.

The adherent cells (HCT-15, HCC827) were each plated a day before the uptake experiment according to the following procedure. 1x poly-L lysine was added into wells of a 96-well plate, and then the plate was incubated at 37 °C for 1 h. The plates were then washed with sterile water and air-dried in tissue culture hood. The adherent cell lines were detached from a plate with 0.25 % trypsin (37°C, 5 min), centrifuged (500g, 5 min; Sorvall Legend, Thermofisher), resuspended in supplemented RPMI, counted using a hemocytometer (Neubauer improved 0.1 mm, Marenfeld, Lauda-Königshofen, Germany), and diluted to 150,000 cells/mL in supplemented RPMI. Aliquots of 100 µL were added into designated wells, and the plate was incubated overnight at 37°C to be ready at the same time as the suspension cells. On the day of the uptake experiment, the media for the adherent cells was changed to 1x HBSS by removing the old media with vacuum and adding the 1x HBSS.

Suspension cells (GS025, GBM39, ParcB3) were each prepared according to the following procedure. 2 mL of cell suspension was dispensed into microcentrifuge tube, the cell clusters were broken apart by pipetting up and down and single cells were counted using a hemocytometer. The concentration was determined and 150,000 cells were aliquoted and centrifuged (500g, 5 min). The supernatant was removed with a pipette and 1x HBSS was added to resuspend the cells to a concentration of 150,000 cells/mL, and then 100 µL aliquots were transferred into designated wells of 96-well filter plate. An additional aliquot was taken for protein concentration estimates.

The [^{18}F]FET was synthesized as described in the main manuscript and formulated in pH 7.4 PBS. The probe was diluted to 370 Bq/µL either in PBS or PBS containing 5 mM FET. After adding 37 kBq (100 µL) of either formulation into the designated culture wells, the well plates were placed into a 37 °C incubator for 10 min.

For the adherent cell plate, the cells were washed with 200 µL cold 1× HBSS (pre-cooled on ice for an hour) three times applying vacuum to remove the media from each well, and lysed with 50 µL of RIPA+ protease inhibitor. The adherent cells were transferred into cold microcentrifuge tubes, placed into gamma counter tubes and counted on a gamma counter for 1 min. For the suspension cells, the cells were washed with cold 1× HBSS three times removing media using vacuum, and the plates were air dried for ~15 min. Wells containing suspension cells were punched into gamma counter tubes and counted on gamma counter for 1 min. After gamma counting, the cell lysate from the adherent cells was stored at -20°C to allow the radioactivity to decay. Protein concentration was determined using the BCA assay protocol the following day. For adherent cells each of the frozen samples was used to measure protein mass directly, and for suspension cells an aliquot taken before the uptake experiment was used for analysis.

4 Preparation of samples with different molar activities

Samples of [^{18}F]FET with different molar activities were prepared from a single batch by dividing the batch and then spiking in different amounts of the reference standard. The molar activity of the batch was measured, then the batch was formulated in 100 µL of sterile saline and divided into 4 portions. The needed amounts of FET reference standard (prepared in stock solutions of 50 µM, 100 µM or 5 mM) to achieve the desired molar activities were then calculated, added, and then the volumes were topped off with additional sterile saline to achieve a final concentration of 3 MBq/100 µL. In parallel with imaging, portions of each sample were re-analyzed via radio-HPLC to measure the final molar activity. Summaries of preparations for two experiments, starting with batches of [^{18}F]FET having molar activities of 35.5 GBq/µmol and 48.1 GBq/µmol, are shown in **Tables S1** and **S2**, respectively**.**

[^{18}F]FET activity, MBq	[^{18}F]FET volume, µL	FET standard concentration, µM	FET standard added volume, µL	Saline volume, µL	Final molar activity, GBq/µmol
8.9	25	–	0	231	35.5
8.9	25	50	5.8	225	10.0
8.9	25	50	26	205	4.81
8.9	25	50	85	146	1.48

Table S1: Preparation of [^{18}F]FET with different molar activities (Experiment 1).

Sample activity, MBq	Activity added volume, µL	Standard concentration, µM	Standard added volume, µL	Saline added volume, µL	Final molar activity, GBq/µmol
8.7	20	0	0	215	48.1
8.7	20	100	3	212	4.81
7.8	66	100	26	141	1.85
7.8	66	5000	3	164	0.37

Table S2: Preparation of [^{18}F]FET with different molar activities (Experiment 2).

The table below summarizes injected activity for each mouse with its corresponding molar activity.

Mouse #	1	2	3	4	5	6	7	8
Experiment 1								
Injection activity, MBq	3.0	2.8	2.9	2.9	–	–	–	–
Molar activity, GBq/µmol	36	10.	4.8	1.5	–	–	–	–
Experiment 2								
Injection activity, MBq	1.6	1.6	1.5	1.6	2.7	2.6	2.6	2.1
Molar activity, GBq/µmol	48	48	4.8	4.8	1.9	1.9	0.37	0.37

Table S3: Summary of amount and molar activity of [^{18}F]FET injected into each mouse for the two *in vivo* imaging studies.

5 *In vivo* imaging in HCC827 xenograft mice

The reference ROIs were selected as follows: a spherical ROI (3 mm diameter) inside the lower left ventricle of the heart (blood uptake reference), and a similar shaped ROI within the leg muscle region beside the femur (muscle reference). The tumor ROIs were selected as an ellipsoid to cover the majority of the tumor volume to minimize the effect of inhomogeneous uptake within a single tumor. The whole-body 3D isocontour ROI was selected based on a minimum signal threshold value set to include all PET signal within the mouse body. The injected dose per volume (% ID/cc) was determined by dividing the integrated intensity in the ROI of interest by the integrated intensity in the whole-body ROI and then dividing by the volume of the ROI.

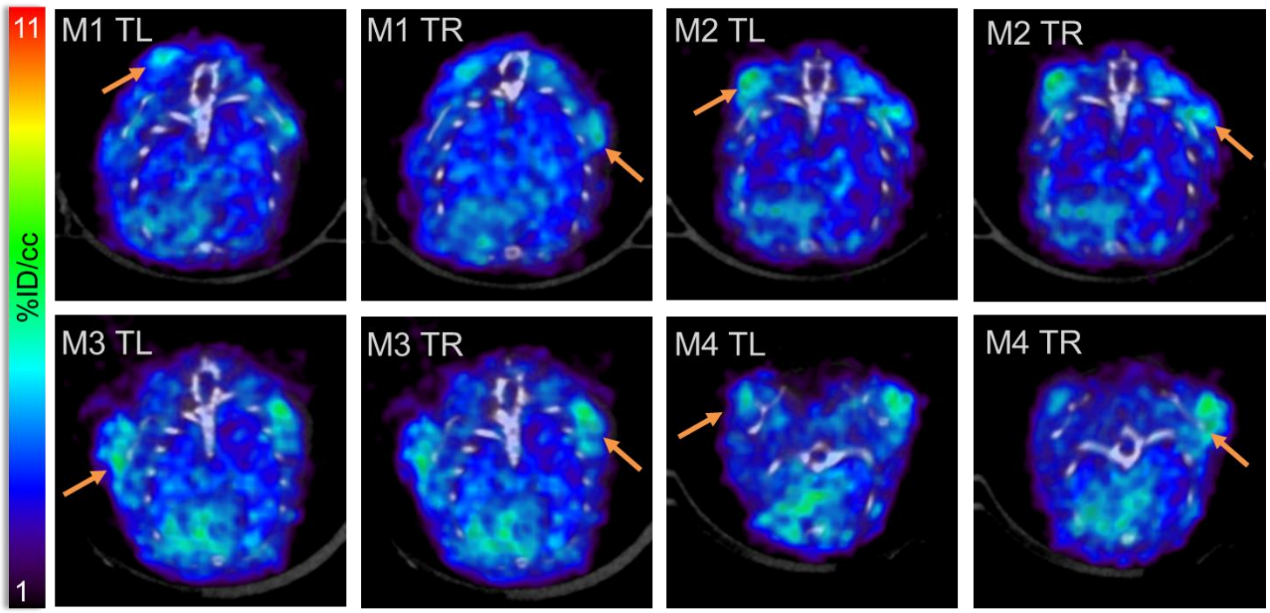

Fig. S3: PET/CT images of each mouse in the first imaging study (36 d post tumor implantation). Each image is a transverse slice through the middle of the indicated tumor, representing a 10 min frame of data 50-60 min post-injection. Each tumor is highlighted by an arrow. M# indicates the mouse number, TL indicates left shoulder tumor, TR indicates right shoulder tumor.

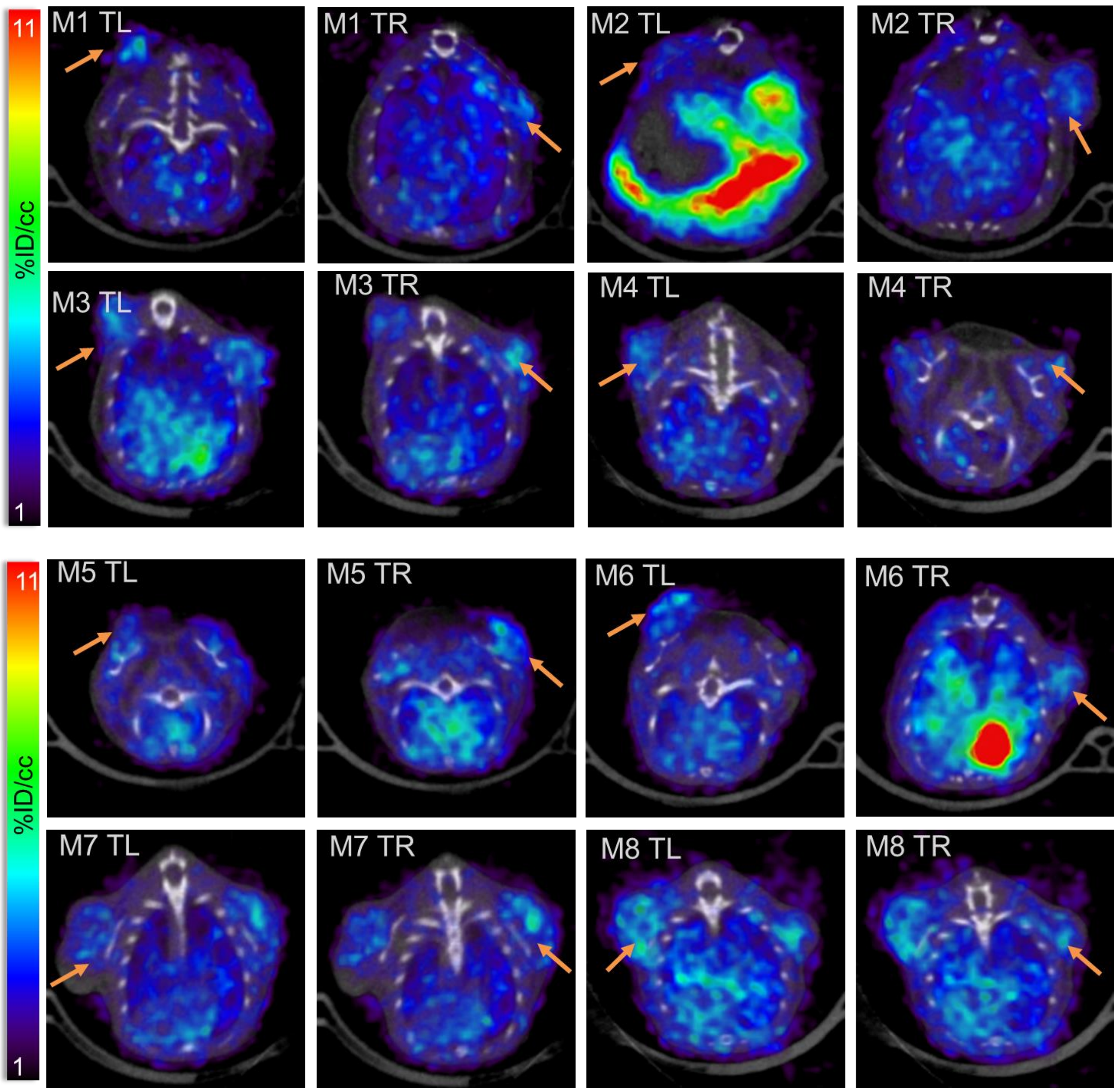

Fig. S4 PET/CT images of each mouse in the second imaging study (50 d post tumor implantation). Each image is a transverse slice through the middle of the indicated tumor, representing a 10 min frame of data 50-60 min post-injection. Each tumor is highlighted by an arrow. M# indicates the mouse number, TL indicates left shoulder tumor, TR indicates right should tumor.

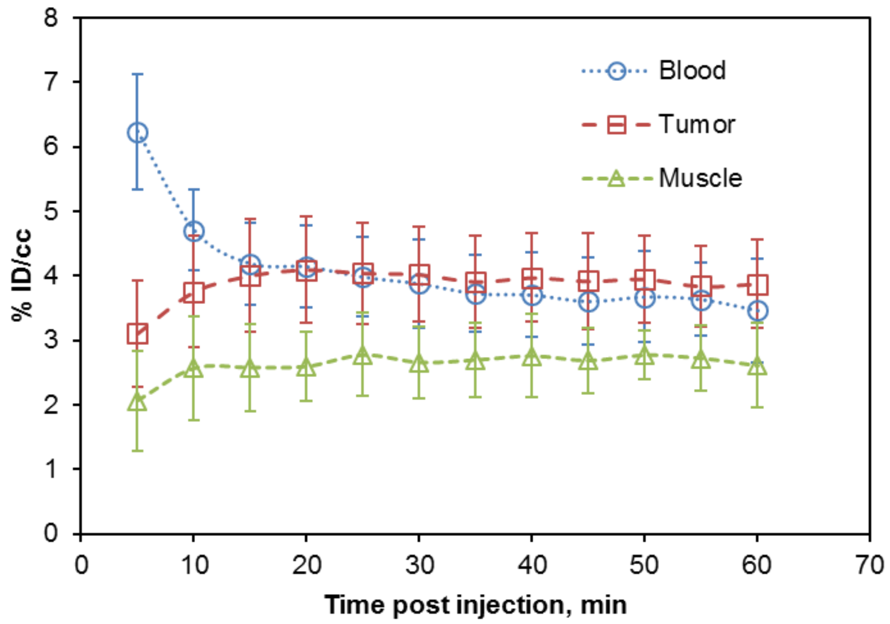

Fig. S5: Uptake of [^{18}F]FET (average value in ROI) for tumor, blood (left ventricle of heart) and muscle (region near femur) is averaged over both imaging studies (i.e. averaged over n = 12 animals for heart and muscle, and averaged over n = 24 for tumors) to show general trends in dynamic biodistribution. Error bars represent standard deviation. %ID/cc is percent of injected dose per cubic centimeter.

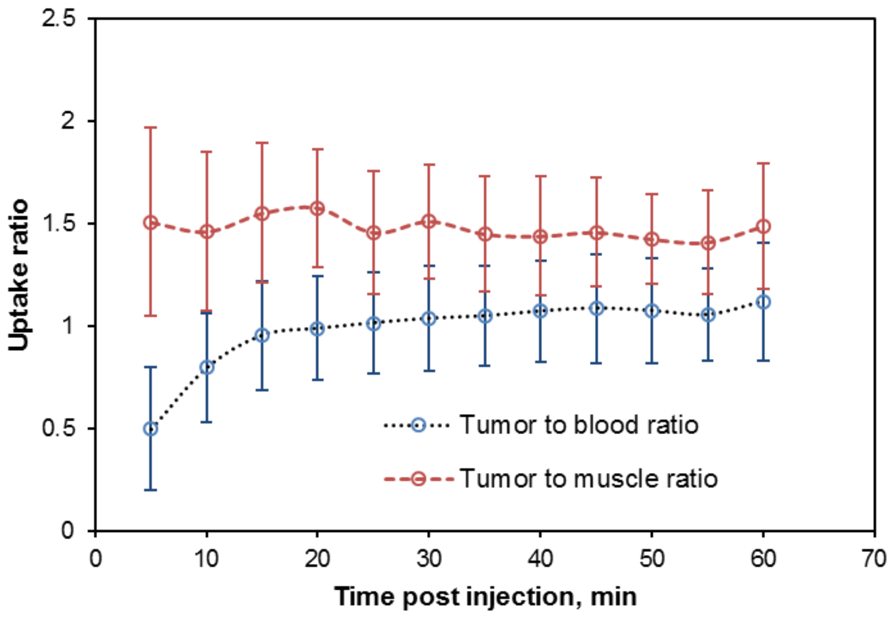

Fig. S6: Tumor to blood and tumor to muscle ratio values averaged over all animals and tumors (i.e. averaged over all molar activity values). Average tumor uptake value across all 12 animals is divided by

either average blood or average muscle uptake. The error bars represent standard deviation determined using error propagation conventions.

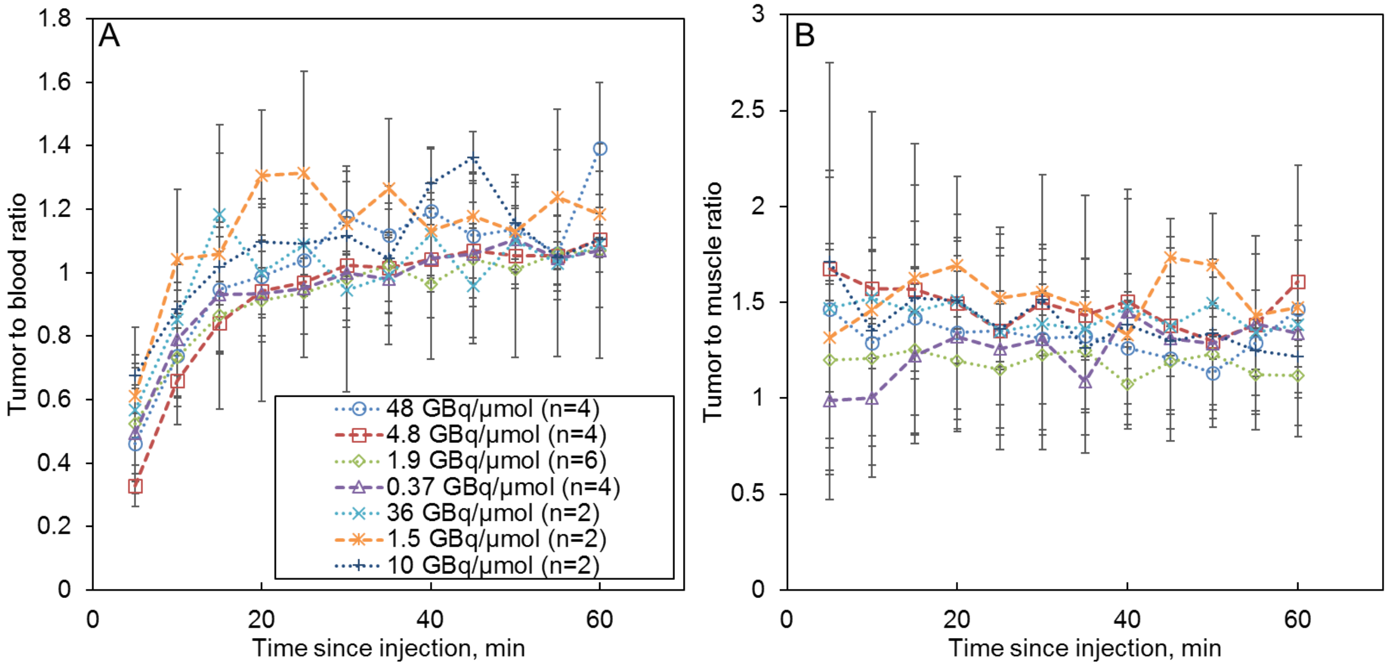

Fig. S7: Summary of dynamic PET imaging. (A) Mean tumor to blood ratio averaged over all tumors with the same molar activity value (n as indicated). (B) Mean tumor to muscle ratio averaged over all tumors with the same molar activity value (n as indicated). The same legend applies to both panels. Error bars represent standard deviations.

6 References

Bourdier T, Greguric I, Roselt P, Jackson T, Faragalla J, Katsifis A. Fully automated one-pot radiosynthesis of O-(2-[18F]fluoroethyl)-l-tyrosine on the TracerLab FXFN module. Nuclear Medicine and Biology. 2011 Jul 1;38(5):645–51.

Iwata R, Pascali C, Terasaki K, Ishikawa Y, Furumoto S, Yanai K. Practical microscale one-pot radiosynthesis of 18F-labeled probes. Journal of Labelled Compounds and Radiopharmaceuticals. 2018;61(7):540–9.

Lakshminarayanan N, Kumar A, Roy S, Pawar Y, Chaudhari PR, Rajan MGR. Fully automated synthesis of O-(2′-[18F]fluoroethyl)-l-tyrosine ([18F]FET) using solid phase extraction (SPE) purification with neutral alumina. J Radioanal Nucl Chem. 2016 Jun 11;1–9.

Ly J, Ha NS, Cheung S, van Dam RM. Toward miniaturized analysis of chemical identity and purity of radiopharmaceuticals via microchip electrophoresis. Anal Bioanal Chem. 2018 Mar 1;410(9):2423–36.
